# Glucose Intolerance and Hyperlipidemia Prior to Diabetes Onset in Female Spontaneously Diabetic Torii (SDT) Rats

**DOI:** 10.1080/15438600490898609

**Published:** 2004

**Authors:** Masami Shinohara, Toshihiro Oikawa, Kahei Sato, Yasunori Kanazawa

**Affiliations:** 1 Department of Pharmacovigilance Torii Pharmaceutical Co., Ltd. 3-4-1, Nihonbashi-Honcho, Chuo-ku Tokyo 103-8439 Japan; 2 Department of Applied Biological Science College of Bioresource Sciences Nihon University Fujisawa Japan; 3 Jichi Medical School Tochigi Japan

## Abstract

The Spontaneously Diabetic Torii (SDT) rat, a newly established animal model for diabetes mellitus, presents nonobese type 2 diabetes with ocular complications. In the present study, oral glucose tolerance tests and biochemical and histopathological examinations were performed in female SDT rats at 16 and/or 25 weeks of age, before the onset of diabetes. At 25 weeks of age, glucose tolerance was significantly impaired, and plasma immunoreactive insulin levels at 120 min after glucose loading were significantly higher (*P* < 0.05). Body weight and fasting levels of plasma triglycerides and nonesterified fatty acids were significantly higher than those in control animals. Histopathologically, inflammatory cell infiltration and fibrosis were observed in and around the pancreatic islets. These results strongly suggest that female SDT rats are useful as a model to investigate impairment of glucose tolerance and hyperlipidemia prior to the onset of diabetes.


*Experimental Diab. Res.*, 5:253–256, 2004
Copyright © Taylor and Francis Inc.
ISSN: 1543-8600 print / 1543-8619 online
DOI: 10.1080/15438600490898609

**Rapid Communications**

### Glucose Intolerance and Hyperlipidemia Prior to Diabetes Onset in Female Spontaneously Diabetic Torii (SDT) Rats

**Masami Shinohara,[1] Toshihiro Oikawa,[1] Kahei Sato,[2] and Yasunori Kanazawa[3]**

[1] *Department of Pharmacovigilance, Torii Pharmaceutical Co., Ltd., Tokyo, Japan*
[2] *Department of Applied Biological Science, College of Bioresource Sciences, Nihon University, Fujisawa, Japan*
[3] *Jichi Medical School, Tochigi, Japan*

**The Spontaneously Diabetic Torii (SDT) rat, a newly established animal model for diabetes mellitus, presents nonobese type 2 diabetes with ocular complications. In the present study, oral glucose tolerance tests and biochemical and histopathological examinations were performed in female SDT rats at 16 and/or 25 weeks of age, before the onset of diabetes. At 25 weeks of age, glucose tolerance was significantly impaired, and plasma immunoreactive insulin levels at 120 min after glucose loading were significantly higher ($P < 0.05$). Body weight and fasting levels of plasma triglycerides and nonesterified fatty acids were significantly higher than those in control animals. Histopathologically, inflammatory cell infiltration and fibrosis were observed in and around the pancreatic islets. These results strongly suggest that female SDT rats are useful as a model to investigate impairment of glucose tolerance and hyperlipidemia prior to the onset of diabetes.**

**Keywords** Animal model; Diabetes; Female SDT Rat; Glucose Intolerance; Hyperlipidemia; Impaired Glucose Tolerance; Pancreatic Fibrosis

### INTRODUCTION

The Spontaneously Diabetic Torii (SDT) rat, a new animal model of nonobese type 2 diabetes, is characterized by the ocular complications (cataracts and retinopathy) [1]. This strain has been used to investigate whether pancreas transplantation (PTx) performed before the "point of no return" prevent/cure diabetic ocular complications [2]. We previously have reported sexual differences in the incidence of diabetes in this strain; glucosuria appeared at 20 weeks of age in males but at 45 weeks of age in females. Although the cumulative incidence of diabetes was 100% at 40 weeks of age in males, it was 33.3% by 65 weeks in females [1]. This higher incidence of diabetes in males also has been reported in several models of spontaneous type 2 diabetes, such as NSY mice[3], TSOD mice [4], Wistar fatty rats [5], Zucker diabetic fatty rats [6], and OLETF rats [7]. The causes of this difference are not fully understood, although sex hormones may play an important role in glucose tolerance [8, 9]. On the other hand, there has been no detailed report on the progression of diabetes in female SDT rats. In the present study, therefore, we performed glucose tolerance tests and biochemical and histopathological examinations to elucidate the process of diabetes in female SDT rats at 16 and/or 25 weeks of age, before the onset of diabetes.

### MATERIALS AND METHODS

### Animals

Female SDT rats were raised at the Yaotsu Branch of the Technical Service Dept, of CLEA Japan, Inc. (Gifu, Japan). Female Sprague-Dawley (SD) rats (Jcl:SD, CLEA Japan, Tokyo, Japan) matched for age were used as control animals. All the rats were maintained in animal facilities under specific pathogen-free conditions where the temperature (20–26°C), humidity (50–70%), and lighting (07:00–19:00 h) were controlled. The

Address correspondence to Masami Shinohara, Department of Pharmacovigilance, Torii Pharmaceutical Co., Ltd., 3-4-1, Nihonbashi-Honcho, Chuo-ku, Tokyo 103-8439, Japan. E-mail: ic2m-snhr@asahi-net.or.jp animals were allowed free access to a commercial pellet (CE-2, CLEA Japan, Tokyo) and sterilized tap water from an automatic water supply system. By 25 weeks of age, the female SDT rats were checked weekly for glucosuria using URIACE (TERUMO, Tokyo), and diagnosed as being diabetic if the urinary glucose was 3+ or higher.

### Oral Glucose Tolerance Test

At 16 and 25 weeks of age, female SDT and control SD rats were subjected to an oral glucose tolerance test (OGTT) after 18 h of fasting. Glucose 2 g/kg body weight was administered by gavage, and blood samples were collected from a tail vein without anesthesia at 0 (preoral administration of glucose), 60 and 120 min to measure plasma glucose and insulin. Plasma glucose was measured by the hexokinase method using COBAS MIRA Plus (Roche, Tokyo). Plasma insulin was measured using an enzyme-linked immunosorbent assay (ELISA) kit (rat insulin kit, Wako, Osaka, Japan). Rats with >185 mg/dl of plasma glucose at 120 min after the administration of glucose were diagnosed as having impaired glucose tolerance (IGT).

### Clinical Examinations and Biochemical Analysis

At 25 weeks of age, female SDT and control SD rats were placed in metabolic cages and after an 18-h fast, all the animals were anesthetized with ether, weighed, and exsanguinated to death. Blood samples were drawn from the abdominal aorta using a heparinized syringe. The heparinized blood samples were centrifuged at 2,000 rpm for 15 min and the plasma was frozen at –80°C until analysis. The plasma concentrations of cholesterol, triglycerides and nonesterified fatty acids (NEFAs) were measured enzymatically using COBAS MIRA Plus (Roche). Immunoreactive leptin in plasma was determined using an enzyme-linked immunosorbent assay (ELISA) kit (Immuno-Biological Laboratories Co., Gunma, Japan).

### Histopathological Examinations

The pancreas from 25-week-old female SDT rats was fixed in 10% neutral buffered formalin. The fixed specimens were embedded in paraffin, sectioned at 4 $\mu$m, and stained with hematoxylin and eosin (H&E) for histopathological examination.

### Statistical Analysis

The data are expressed as the mean±SE . Differences between the female SDT and age-matched control SD rats were assessed by Student's $t$-test. Probability values of $P < 0.05$ were considered as denoting statistical significance.

### RESULTS

### Glucose Tolerance

The results of OGTT in 16- and 25-week-old SDT and control SD rats are shown in Table 1. At 16 weeks of age, SDT rats had significantly higher plasma concentrations of glucose than the control rats, both at 0 and 60 min after the administration of glucose. At 25 weeks of age, the plasma concentration of glucose at 0, 60, and 120 min after the administration of glucose was significantly increased in the SDT rats indicating IGT. At 60 min after the administration of glucose, the plasma concentration of insulin was significantly higher in SDT rats 6 weeks of age. At 120 min after the administration of glucose, the concentration of insulin was also significantly higher in SDT rats 25 weeks of age.

### Clinical Examinations and Biochemical Analysis

Glucosuria was not detected in SDT rats 25 weeks of age. The body weight and fasting plasma triglycerides, total cholesterol, NEFA and leptin concentrations in SDT and control SD rats are shown in Table 2. The body weight of SDT rats was significantly higher ($P < 0.05$) than that of control rats. Plasma concentrations of triglycerides and NEFA were almost 4 times

**TABLE 1**
Plasma glucose and insulin responses in female SDT and control SD rats subjected to an oral glucose tolerance test at 16 and 25 weeks of age

| Age in weeks | Strain | No. of animals | Plasma glucose (mg/dl) | | | Plasma insulin (ng/ml) | | |
|---|---|---|---|---|---|---|---|---|
| | | | 0 min | 60 min | 120 min | 0 min | 60 min | 120 min |
| 16 | SD | 6 | 112.8 ± 5.9 | 175.5 ± 7.4 | 161.7 ± 8.6 | 0.49 ± 0.09 | 0.62 ± 0.13 | 0.56 ± 0.1 |
| | SDT | 6 | 130.6 ± 3.4* | 232.6 ± 13.9† | 159.3 ± 8.5 | 0.62 ± 0.03 | 1.21 ± 0.16* | 0.74 ± 0.1 |
| 25 | SD | 6 | 124.9 ± 7.2 | 180.7 ± 8.7 | 167.9 ± 7.4 | 1.19 ± 0.24 | 1.25 ± 0.24 | 1.21 ± 0.14 |
| | SDT | 6 | 145.1 ± 4.3* | 312.0 ± 44.6* | 253.9 ± 19.2† | 1.17 ± 0.13 | 1.54 ± 0.11 | 1.64 ± 0.11* |

Values are expressed as the mean ±SE+A1.
*$P < 0.05$ versus the control SD rat group.
†$P < 0.01$versus the control SD rat group.

**TABLE 2**
Body weight and fasting plasma concentrations of triglycerides, total cholesterol, nonesterified free fatty acids and leptin in female SDT and control SD rats at 25 weeks of age

| Parameter | SDT (n = 6) | SD (n = 6) |
|---|---|---|
| Body weight (g) | 367.3 ± 8.1* | 329.3 ± 12.7 |
| Triglycerides (mg/dl) | 91.5 ± 28.1* | 22.7 ± 3.3 |
| Total cholesterol (mg/dl) | 83.3 ± 4.1 | 89.6 ± 4.7 |
| Nonesterified fatty acids (meq/l) | 0.88 ± 0.03† | 0.71 ± 0.03 |
| Leptin (pg/ml) | 24096 ± 5199 | 10107 ± 5712 |

Values are expressed as the mean ±SEM.
*$P < 0.05$ versus the control SD rat group.
†$P < 0.01$versus the control SD rat group.

and 1.2 times higher in SDT rats than in control rats, respectively. On the other hand, the higher concentration of leptin observed in SDT rats was not significantly different from that in control SD rats and there were no differences between the two groups regarding their plasma total cholesterol.

### Histopathological Findings

The histopathological features of the pancreas in female SDT rats are shown in Figure 1. At 25 weeks of age, hemosiderin deposition, infiltration of lymphocytes and macrophages, and fibrous tissue proliferation in and around the pancreatic islets were observed and islets were divided into small lobules by fibrous tissue. These histopathologic changes were qualitatively similar to those observed in male SDT rats [1].

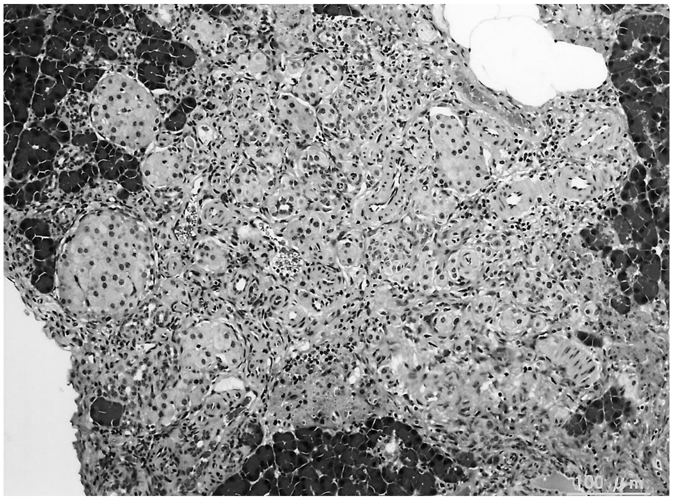

**FIGURE 1**
Histopathological changes in the pancreas of a female SDT rat at 25 weeks of age. Inflammatory cell infiltration and fibrosis around the pancreatic islets are observed (H&E stain, ×140).

### DISCUSSION

The characteristics of male SDT rats are glucose intolerance with impaired insulin secretion and lesions in pancreatic islets, including hemorrhage and inflammatory cell infiltration, at the prediabetic stage [1, 10]. In male SDT rats, using the quantitative trait locus (QTL) analysis, we identified three highly significant QTLs (*Giadt1*, *Gisdt2*, and *Gisdt3*) for glucose intolerance on rat chromosomes 1, 2, and X, respectively [11]. In this investigation, we confirmed that glucose intolerance and pancreatic lesions developed prior to clinical diabetes mellitus in female SDT rats, the same as in male SDT rats. Therefore, the glucose-intolerance-related loci involved in male SDT rats were thought to be also strongly involved in the development of diabetes in females.

Although there are some differences in the insulin pattern between males and females [10], the plasma concentrations of insulin in female SDT rats was higher than that in age-matched control rats during OGTT. This fact suggested that an insulin-resistant factor or other factors causing an enhancement of insulin requirements might have been present in the preclinical stage. Obesity is a well-known factor of insulin resistance [12], but in the present study, as previously reported for males, obesity was not detected. In this study, we also found a significant increase of fasting plasma triglycerides and NEFA at the prediabetic stage. In humans, the increase of VLDL-triglycerides concentration is closely related to an impairment in the ability of insulin to suppress NEFAs [13, 14]. In addition, it has been reported that in 6-week-old OLETF rats, in which insulin resistance had not been manifested, visceral fat weight was already higher and free fatty acids (FFAs) and VLDL-triglycerides were elevated, compared with the control rats [15]. In order to clarify the causal factors of the increased plasma insulin concentration during OGTT in female SDT rats, further investigations (e.g., measurement of visceral fat volume) should be performed.

In the present study, it becomes clear that female SDT rats suffered from IGT at 25 weeks of age when they had not developed overt diabetes yet. It seems likely that most female SDT rats present IGT for a long time and do not develop diabetes. According to the diagnostic criteria established by the World Health Organization in 1998, other categories of hyperglycemia included IGT and impaired fasting glycemia (IFG) [16]. Kadowaki et al. [17] reported that 2% of patients with IGT proceeded annually to diabetes mellitus in Japan. Furthermore, IGT attracts great attention as an independent risk factor for coronary heart disease [18,19].

In conclusion, further examination of long-term IGT in female SDT rats would lead to the clarification of factors involved in the transition from IGT to diabetes and prevention of IGT.

### REFERENCES

[1] Shinohara, M., Masuyama, T., Shoda, T., Takahashi, T., Katsuda, Y., Komeda, K., Kuroki, M., Kakehashi, A., and Kanazawa, Y. (2000) A new spontaneously diabetic non-obese Torii rat strain with severe ocular complications. *Int. J. Exp. Diab. Res.*, **1**, 89–100.

[2] Miao, G., Ito, T., Uchikoshi, F., Kamei, M., Akamaru, Y., Kiyomoto, T., Komoda, H., Nozawa, M., and Matsuda, H. (2004) Stage-dependent effect of pancreatic transplantation on diabetic ocular complications in the spontaneously diabetic torii rat. *Transplantation*, **77**, 658–663.

[3] Ueda, H., Ikegami, H., Yamato, E., Fu, J., Fukuda, M., Shen, G., Kawaguchi, Y., Takekawa, K., Fujioka, Y., Fujisawa, T., Nakagawa, Y., Hamada, Y., Shibata, M., and Ogihara, T. (1995) The NSY mice: A new animal model of spontaneous NIDDM with moderate obesity. *Diabetologia*, **38**, 503–508.

[4] Suzuki, W., Iizuka, S., Tabuchi, M., Funo, S., Yanagisawa, T., Kimura, M., Sato, T., Endo, T., and Kawamura, H. (1999) A new mouse model of spontaneous diabetes derived from ddY strain. *Exp. Anim.*, **48**, 181–189.

[5] Ikeda, H., Shino, A., Matsuo, T., Iwatsuka, H., and Suzuoki, Z. (1981) A new genetically obese-hyperglycemic rat (Wistar Fatty). *Diabetes*, **30**, 1045–1050.

[6] Clark, J. B., Palmer, C. J., and Shaw, W. N. (1983) The diabetic Zucker fatty rat. *Soc. Exp. Biol. Med.*, **173**, 68–75.

[7] Kawano, K., Hirashima, T., Mori, S., Saitoh, Y., Kurosumi, M., and Natori, T. (1992) Spontaneous long-term hyperglycemic rat with diabetic complications, *Diabetes*, **41**, 1422–1428.

[8] Shi, K., Mizuno, A., Sano, T., Ishida, K., and Shima, K. (1994) Sexual difference in the incidence of diabetes mellitus in Otsuka-Long-Evans- Tokushima-Fatty rats: Effects of castration and sex hormone replacement on its incidence. *Metabolism*, **43**, 1214–1220.

[9] Tarres, M. C., Martinez, S. M., Montenegro, S. M., Figueroa, N. S., D'Ottavio, A. E., and Picena, J. C. (1997) Influence of gonadectomy in eSS diabetic rats. *J. Physiol. Biochem.*, **53**, 211–216.

[10] Masuyama, T., Komeda, K., Hara, A., Noda, M., Shinohara, M., Oikawa, T., Kanazawa, Y., and Taniguchi, K. (2004) Chronological characterization of diabetes development in male spontaneously diabetic Torii rats. *Biochem. Biophys. Res. Commun*, **314**, 870–877.

[11] Masuyama, T., Fuse, M., Yokoi, N., Shinohara, M., Tsujii, H., Kanazawa, M., Kanazawa, Y., Komeda, K., and Taniguchi, K. (2003) Genetic analysis for diabetes in a new rat model of nonobese type 2 diabetes, spontaneously diabetic Torii rat. *Biochem. Biophys. Res. Commun.*, **304**, 196–206.

[12] Moller, D. E., and Flier, J. S. (1991) Insulin resistance-mechanisms, syndromes, and implications. *N. Engl. J. Med.*, **325**, 938–948.

[13] Reaven, G. M., and Greenfield, M. S. (1981) Diabetic hypertriglyceridemia: Evidence for three clinical syndromes. *Diabetes*, **30**(suppl 2), 66–75.

[14] Yki-Jarvinen, H., and Taskinen, M-R. (1988) Interrelationships among insulin's antilipolytic and glucoregulatory effects and plasma triglycerides in nondiabetic and diabetic patients with endogenous hypertriglyceridemia. *Diabetes*, **37**, 1271–1278.

[15] Kuriyama, H., Yamashita, S., Shimomura, I., Funahashi, T., Ishigami, M., Aragane, K., Miyaoka, K., Nakamura, T., Takemura, K., Man, Z., Toide, K., Nakayama, N., Fukuda, Y., Lin, M. C. L., Wetterau, J. R., and Matsuzawa, Y. (1998) Enhanced expression of hepatic acyl-coenzyme A synthetase and microsomal RNAs in the obese and hypertriglyceridemic rat with visceral fat accumulation. *Hepatology*, **27**. 557–562.

[16] Alberti, K. G. M. M., and Zimmet, P. Z. (for the WHO Consultation). (1998) Definition, diagnosis and classification of diabetes mellitus and its complications. Part 1: Diagnosis and classification of diabetes mellitus. Provisional report of a WHO Consultation. *Diab. Med.*, **15**, 539–553.

[17] Kadowaki, T., Miyake, Y., Hagura, R., Akanuma, Y., Kajinuma, H., Kazuya, N., Takaku, F., and Kosaka, K. (1984) Risk factors for worsening to diabetes in subjects with impaired glucose tolerance. *Diabetologia*, **26**, 44–49.

[18] Yamasaki, Y., Kawamori, R., Mastushima, H., Nishizawa, H., Kodama, M., Kubota, M., Kajimoto, Y., and Kamada, T. (1995) Asymptomatic hyperglycaemia is associated with increased intimal plus medial thickness of the carotid artery. *Diabetologia*, **38**, 585–591.

[19] The DECODE study group, on behalf of the European diabetes epidemiology group. (2001) Glucose tolerance and cardiovascular mortality: Comparison of fasting and 2-hour diagnostic criteria. *Arch. Intern. Med.*, **161**, 397–405.

